# Chinese Herbal Medicine for Symptom Management in Cancer Palliative Care: Systematic Review And Meta-analysis: Erratum

**DOI:** 10.1097/01.md.0000484108.92666.50

**Published:** 2016-05-20

In the article “Chinese Herbal Medicine for Symptom Management in Cancer Palliative Care: Systematic Review And Meta-analysis”,^1^ which appeared in Volume 95, Issue 7 of *Medicine*, Dr. Ng's degree was listed incorrectly. The article has since been corrected online.
